# Supplementary material for: Mucoadhesive In Situ Rectal Gel Loaded with Rifampicin: Strategy to Improve Bioavailability and Alleviate Liver Toxicity
Source: Pharmaceutics. 2021 Mar 5;13(3):336. doi: 10.3390/pharmaceutics13030336 (PMC8001001; doi:10.3390/pharmaceutics13030336)
Supplement: Supplementary file 1 [file pharmaceutics-13-00336-s001.pdf]

pharmaceutics

Supplementary Materials: Mucoadhesive In Situ Rectal Gel Loaded with Rifampicin: Strategy to Improve Bioavailability and Alleviate Liver Toxicity

Fakhria Al-Joufi, Mohammed Elmowafy, Nabil K. Alruwaili, Khalid S. Alharbi, Khaled Shalaby, Shaker Alshararee and Hazim M. Ali

Citation: Al-Joufi, F.; Elmowafy, M.; Alruwaili, N.K.; Alharbi, K.S.; Shalaby, K.; Ali, H.M. Mucoadhesive In Situ Rectal Gel Loaded with Rifampicin: Strategy to Improve Bioavailability and Alleviate Liver Toxicity. *Pharmaceutics* **2021**, *13*, 336. https://doi.org/10.3390/pharmaceutics13030336

Academic editor: Nihal Engin Vrana

Received: 25 January 2021
Accepted: 1 March 2021
Published: 5 March 2021

Publisher's Note: MDPI stays neutral with regard to jurisdictional claims in published maps and institutional affiliations.

Copyright: © 2021 by the authors. Licensee MDPI, Basel, Switzerland. This article is an open access article distributed under the terms and conditions of the Creative Commons Attribution (CC BY) license (http://creativecommons.org/licenses/by/4.0/).

1. Method Validation

The optimized method for quantification of RF has been validated for evaluating linearity, limit of detection (LOD), limit of quantitation (LOQ), precision, and accuracy. The linearity was determined with seventeen concentration levels (0.05, 0.1, 0.3, 0.6, 1, 3, 5, 7, 10, 15, 20, 30, 40, 50, 60, 70, 80 μg/mL) of RF. LOD and LOQ of RF were calculated from linear regression equation based on the slope and SD of the intercept using the following formula: LOD = 3.3σ/S and LOQ = 10 σ/S, where σ is the standard deviation of intercept and S is the slope of the calibration curve. Precision and accuracy were evaluated at two levels of RF (3 and 15 μg/mL) and were assessed by calculating the relative standard deviation (RSD %= (σ/ mean measured concentration) × 100, where σ is the standard deviation of intercept) and the percentage recovery (R %= [obtained concentration] / [added concentration] x 100) respectively of two concentration levels in triplicates. The system suitability was evaluated by estimating retention time, peak area, theoretical plates and peak asymmetry for 5 μg/mL of RF.

2. Chromatography

2.1. Optimization of Chromatographic Conditions

Different factors such as detection wavelength, constitution and proportion of the mobile phase, and other elements that influence the separation of RF were evaluated. Different wavelengths were tested and the 249 nm wavelength showed good sensitivity and resolution and thus selected for determination of rifampicin. The column types (C8 and C18), different mobile phases, the concentration of formic acid, the proportion of mobile phase, and flow rate were tested to improve and facilities the separation and get reasonable retention time. As a result, the separation was accomplished using a C18 column and isocratic elution with a mobile phase consisted of the mixture of acetonitrile (solvent A) and 0.01 % formic acid in water (solvent B) (75: 25 *v*/*v*) at a flow rate of 0.8 ml/min. Based on the above mentioned, RF peak was observed at the retention time of 3.433 min as shown in Figure S1A.

2.2. Validation of Method

2.2.1. Linearity

Under optimum condition, a linear relationship was tested by plotting peak area versus rifampicin standard solutions (Figure S1B). Linearity of the calibration curve was obtained in the range of 0.05–80 μg/mL with good correlation coefficients (r) of 0.9994 and linear regression equation was found to be; Area = 0.437C − 0.232.

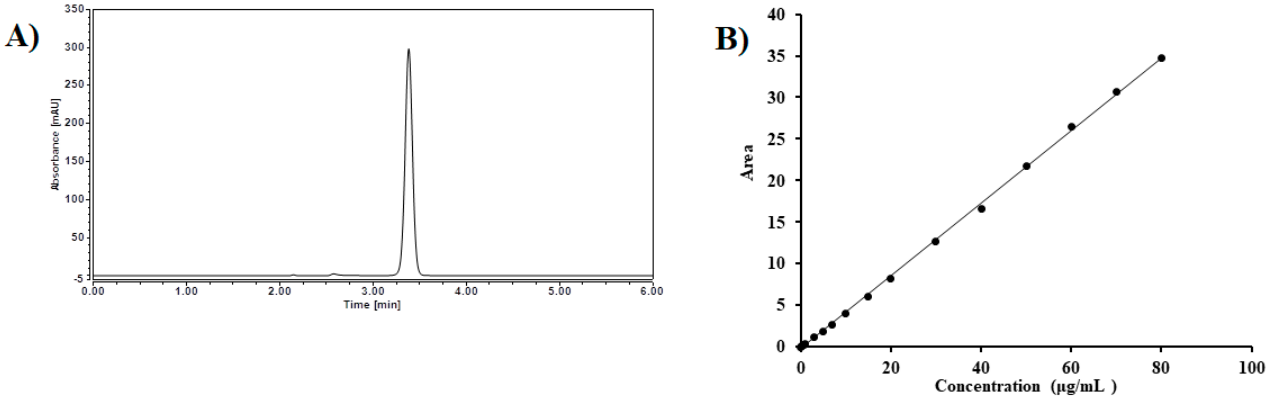

Figure S1. Chromatography of RF determination; (**A**) HPLC-DAD chromatogram of 5 μg/mL of RF and (**B**) analytical calibration curve for RF.

2.2.2. LOD and LOQ

LOD and LOQ of RF were calculated according to the formula mentioned above and found to be 0.011 and 0.034 μg/mL respectively, which indicated the high sensitivity of the method.

2.2.3. Accuracy and Precision

Accuracy and precision of the proposed analytical method were evaluated by injection of two RF concentration levels (3 and 15 μg/mL) in triplicates. The accuracy and precision values, were calculated according to formulas mentioned above, are shown in Table S1. The results for both intraday and inter-day determinations indicated good accuracy and precision of the developed method for the quantification of RF.

Table S1. Accuracy and precision of the proposed method for the determination of RF.

RF Concentration (μg/ml)	Intra-Day				Inter-Day			
	Average Concentration Found (μg/mL)	Standard Deviation	Recovery (%)	RSD (%)	Average Concentration Found (μg/mL)	Standard Deviation	Recovery (%)	RSD (%)
3	2.99	0.021	99.67	0.7	2.97	0.01	99	0.34
15	14.25	0.11	95	0.77	14.19	0.08	94.6	0.56

2.2.4. System suitability

The peak retention time, peak area, peak asymmetry and number of theoretical plates as parameters of system suitability were calculated at 5 μg/mL of rifampicin in triplicates. The results of the determination of peak retention time, peak area, peak asymmetry and number of theoretical plates were found to be 3.38± 0.003, 1.862± 0.006, 0.953± 0.005 and 3830± 54.11 respectively. These values designated good selectivity of method.
